# Effect of sequence and metal ions on UVB-induced *anti* cyclobutane pyrimidine dimer formation in human telomeric DNA sequences

**DOI:** 10.1093/nar/gku163

**Published:** 2014-03-04

**Authors:** Jillian E. Smith, Chen Lu, John-Stephen Taylor

**Affiliations:** Department of Chemistry, Washington University, St. Louis, MO 63130, USA

## Abstract

Irradiation of G-quadruplex forming human telomeric DNA with ultraviolet B (UVB) light results in the formation of *anti* cyclobutane pyrimidine dimers (CPDs) between loop 1 and loop 3 in the presence of potassium ions but not sodium ions. This was unexpected because the sequences involved favor the nonphotoreactive hybrid conformations in K^+^ solution, whereas a potentially photoreactive basket conformation is favored in Na^+^ solution. To account for these contradictory results, it was proposed that the loops are too far apart in the basket conformation in Na^+^ solution but close enough in a two G-tetrad basket-like form 3 conformation that can form in K^+^ solution. In the current study, Na^+^ was still found to inhibit *anti* CPD formation in sequences designed to stabilize the form 3 conformation. Furthermore, *anti* CPD formation in K^+^ solution was slower for the sequence previously shown to exist primarily in the proposed photoreactive form 3 conformation than the sequence shown to exist primarily in a nonphotoreactive hybrid conformation. These results suggest that the form 3 conformation is not the principal photoreactive conformation, and that G-quadruplexes in K^+^ solution are dynamic and able to access photoreactive conformations more easily than in Na^+^ solution.

## INTRODUCTION

Telomeres are the capping structures at the ends of each chromosome that are responsible for protecting DNA against gene erosion during cellular division and act as a cellular marker for apoptosis when the sequence has been shortened to a critical length (1). Human telomeres consist of several hundred to thousands of noncoding repeats of the sequence TTAGGG with a 100–200-nt single-stranded 3′-end that folds back to form a strand displacement loop with a complementary section of the telomeric DNA. The displaced oligomeric TTAGGG strand is bound by a six-protein shelterin complex composed of the telomere repeat binding factors TRF1, TRF2 and protection of telomere protein POT1, along with accessory proteins RAP1, TPP1 and TIN2a (2,3). These proteins serve to protect the otherwise single-stranded telomeric DNA from degradation.

**Figure 1.**
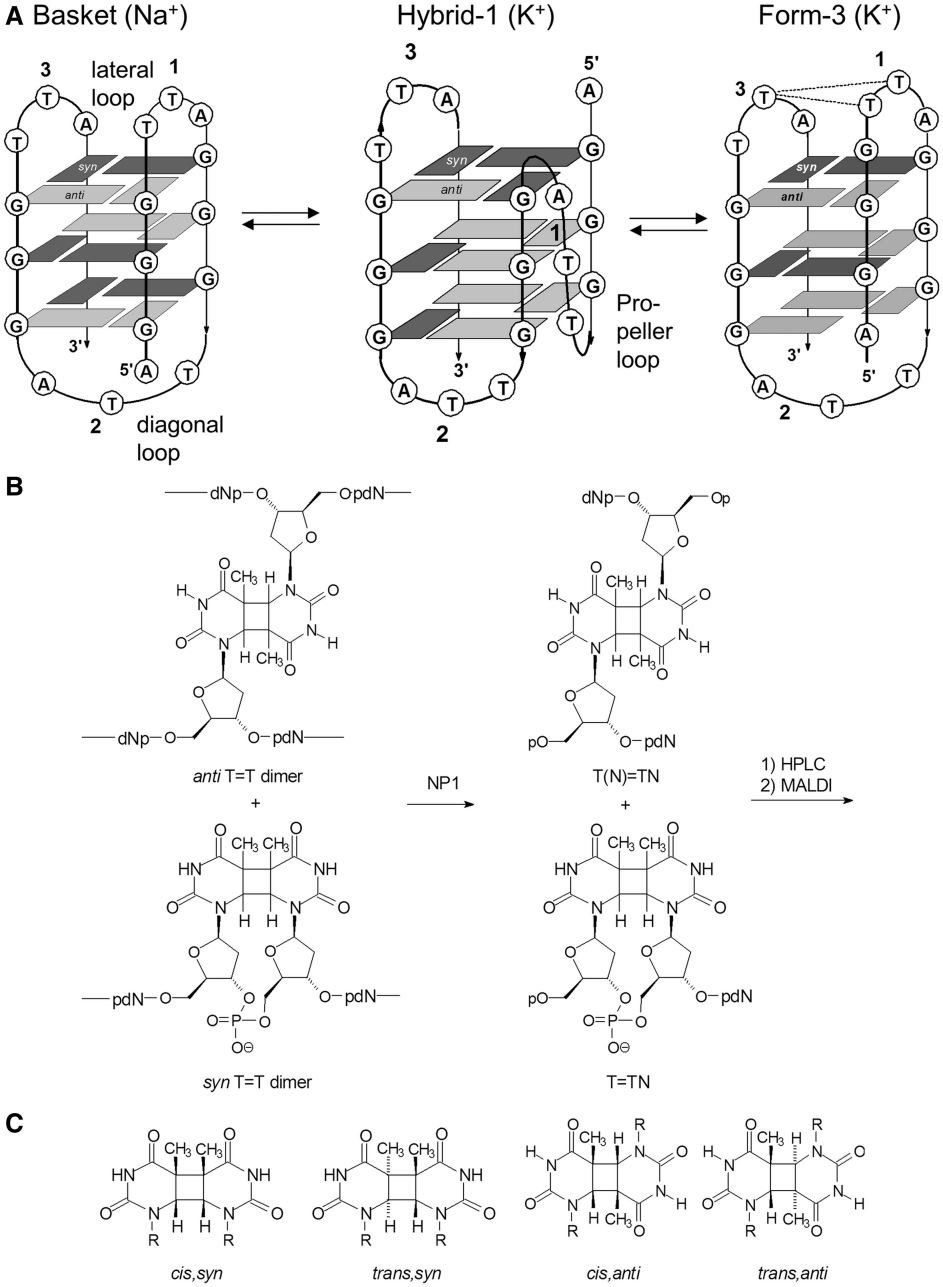
DNA photoproduct formation in human telomeric G-quadruplexes. (**A**) The basket form has been detected in Na^+^ solution, whereas the hybrid-1 structure and two-tetrad basket-type form 3 structure have been detected in K^+^ solutions. The hybrid-2 structure is similar to the hybrid-1 structure except that loop 1 and 2 are lateral and loop 3 is propeller. The dashed lines indicate the pairs of T's that form the *anti* CPDs. (**B**) Structure of *syn* and *anti* type CPDs and their detection and quantification by a combined nuclease P1 coupled HPLC/mass spectrometry assay. (**C**) Stereochemistry of the cyclobutane ring portion of the CPDs.

In solution, short fragments of the single-stranded G-rich human telomeric sequence fold to form a number of different quadruplex forms, such as chairs, baskets and hybrid structures, that are stabilized by a cyclic planar array of four Gs that interact via Hoogsteen hydrogen bonding called G-quartets. The specific structure that a sequence adopts depends on the repeat length, cation and the length and sequence of the 5′ and 3′ tails (4–8). For example, it has been found from nuclear magnetic resonance (NMR) studies that the oligonucleotide d[AGGG(TTAGGG)_3_], (Tel22), principally forms a basket structure in the presence of sodium ion (9), but converts to a mixture of hybrid-1 and hybrid-2 structures that have no adjacent lateral loops in the presence of potassium ion (10) (Figure 1A). On the other hand, the sequence d[AAAGGG(TTAGGG)_3_AA], (Tel26), which initially forms a mixture of hybrid-1 and hybrid-2 structures in the presence of K^+^, converts primarily to the hybrid-1 conformer (10,11). Recently, another unique G-quadruplex structure, form 3, has been discovered for d[GGG(TTAGGG)_3_T], (NF3), in K^+^ solution that forms a basket-type structure that contains only two G quartets instead of three (12). The formation of only two quartets results from the slipping of the first G-strand of the basket form up by 1 nt to form a 4-nt GTTA loop instead of the 3-nt loop found in the basket form. The form 3 structure is further stabilized by capping structures on either end of the two-tetrad core that involves extensive interactions between the proximal loops on one side and between the 5′ and 3′ ends on the other side. The two-tetrad G-quadruplex conformation has also been suggested as an intermediate in the interconversion between hybrid-1 and -2 structures for a related sequence in which a G was changed to an I to disrupt quartet formation (13).

It is not known with certainty which of the possible conformations of G-quartet forming sequences is present as stable structures in human telomeres *in vivo*, or may form transiently during replication and repair. The length of the human telomeric single-stranded overhang can be as long as 300 nt, thus containing many potential sites for single and multiple G-quadruplex structures to form. The bead-on-a-string model for long telomeric sequences suggests that each G-quadruplex folds independently of its neighboring G-quadruplex and they are then linked together by short segments of single-stranded DNA (14). In dilute solutions of K^+^-containing buffers, hybrid conformers are the major G-quadruplex structures (15,16), but may contain G-quadruplexes with other structural motifs (17). A recent report has also provided *in vitro* and *in vivo* high-resolution NMR evidence that the quadruplex structures equilibrate between the hybrid-1 and two-tetrad form 3 basket structures, and that the form 3 structure is actually the dominant species (18).

Recently, through the use of an enzyme-coupled mass spectrometry assay, cyclobutane pyrimidine dimers (CPDs) with an unusual *anti* stereochemistry (Figure 1B) were found to form between thymines in loop 1 and loop 3 of Tel22 when irradiated with UVB light in K^+^ solution, with the *trans,anti* T(A)=T(A) CPD predominating (19) (See Figure 1C for the designation of the stereoisomers). For the *anti* CPD to have formed, the two Ts must have been able to stack upon each other, which would rule out the involvement of the hybrid-1 and -2 structures previously proposed for these sequences in K^+^ solution (10). Equally, if not more surprising, the *anti* CPD did not form in Na^+^ solution, in which Tel22 adopts the basket structure that had initially been predicted to facilitate *anti* CPD formation between adjacent lateral loops 1 and 3.

To account for the unexpected photochemical behavior of Tel22 in Na^+^ and K^+^ solutions, it was proposed that Tel22 adopts the basket-like form 3 structure (Figure 1A) in K^+^ solution, which, because of the larger 4-nt loop 1, enables the Ts in loops 1 and 3 to stack on each other and form the *anti* CPD on UVB irradiation. The failure to form *anti* CPDs in Na^+^ solution could then be explained by Na^+^ stabilization of the basket structure with the smaller 3-nt loop 1 of the basket structure, which would not enable the Ts to stack on each other and photodimerize. Deleting the 5′-AGG or changing the first three Gs of the Tel22 sequence to As to prevent quadruplex formation, resulted in a greatly diminished yield of *anti* CPDs with much less stereoselectivity in K^+^ solution (19). Irradiation of the Tel26 sequence, which was found to almost exclusively adopt the hybrid-1 structure by NMR, produced almost exclusively the *trans,anti* CPD of T(A)=T(A). Therefore, the stereoselective formation of the *trans,anti* CPD of T(A)=T(A) in both Tel22 and Tel26 in K^+^ solution suggested there must be a significant amount of a photoreactive conformation in equilibrium with the hybrid structures, which was presumed to be the form 3 structure.

In this report, we have attempted to obtain additional evidence for the involvement of a two-tetrad form 3 basket-type conformation in UVB-induced *anti* CPD formation in human telomeric DNA through site-specific mutations of the Tel26 and NF3 sequences meant to stabilize a 4-nt loop that is present in this conformation. We also investigated the effects of sequence and sodium and potassium ions on the T_m_, circular dichroism spectra and *anti* CPD formation. We find that *anti* CPD formation in all the sequences was suppressed relative to the *syn* CPD by sodium ion, while its formation in the presence of potassium ion depended on sequence and temperature. Most surprisingly, the Tel26 sequence, which is mainly in a nonphotoreactive hybrid structure, photoreacted to give the *trans,anti* CPD at a greater rate than the NF3 sequence, which is supposed to exist primarily in the proposed photoreactive conformation. These results indicate that some other conformation is involved in forming the *trans,anti* CPD, and that human telomeric G-quadruplex forming sequences are more dynamic in K^+^, and can freely equilibrate between nonphotoreactive and photoreactive conformations. These results also suggest that *anti* CPDs are not restricted to canonical human telomeric sequences and might also form in other G-quadruplex-forming sequences, such as those found in promoters.

## MATERIALS AND METHODS

### Materials

Oligodeoxynucleotides (ODNs) were purchased from Integrated DNA Technologies, Inc. (IDT) (Coralville, IA, USA). Nuclease P1 (NP1) from *Penicillium citrinum* was obtained from Sigma (St. Louis, MO, USA). Ammonium citrate and 3-hydroxypicolinic acid for use as matrix assisted laser desorption ionization (MALDI) matrices were purchased from Fluka (Milwaukee, WI, USA). High-performance liquid chromatography (HPLC) solvents were from Fisher (Fair Lawn, NJ, USA).

### Instrumentation

HPLC separation and analysis were carried out on System Gold HPLC BioEssential with a binary gradient Model 125 pump and a Model 168 diode array detector (Beckman Coulter, Inc., Fullerton, CA, USA). An X-Bridge column (C18, 4.6 × 75 mm, 2.5 μm, 135 Å) from Waters Corporation (Milford, MA, USA) was used for reverse-phase HPLC. UVB (280–320 nm) irradiation was carried out with two 15-W Spectroline BLE-IT158 broadband UVB tubes with peak UV (312 nm) intensity of 1150 μW/cm^2^ at 25 cm, and a Longlife filter glass from Spectronics Corporation (Westbury, NY, USA). MALDI mass spectra (MS) were collected on an Applied Biosystems 4700 tandem time-of-flight mass spectrometer (Applied Biosystems, Foster City, CA, USA). Circular dichroism (CD) experiments were carried out on a J-810 spectropolarimeter (Jasco). UV melting curves were obtained on a Cary 100 Bio UV-VIS Spectrometer (Varian).

### Preparation of G-quadruplexes

Unless otherwise stated, the ODNs (50 µM) in 10 mM Tris-HCl, pH 7.5, with 150 mM NaCl or KCl were heated at 95°C for 10 min and then rapidly cooled in ice. Formation of G-quadruplexes was confirmed by CD.

### Circular dichroism

CD spectra were acquired immediately after sample preparation at 4°C or room temperature or after storage of up to several days at 4°C. The measurements were carried out with 250 μl of 50 μM ODN samples in a 1-mm path quartz cell under nitrogen. Spectra shown were the average of three accumulations from 220 to 320 nm with a band width of 1 nm, response time of 1 s, data pitch of 0.2 nm and a scan speed of 50 nm/min.

### Melting temperature experiments

ODN samples (100 μl of 50 µM) in 10 mM Tris, 150 mM KCl, pH 7.4, buffer were preheated in cuvette to 95°C for 10 min, and any air bubbles that formed were removed by tapping the cuvette. The absorbance was recorded at 295 nm to detect quadruplex formation (20), and the initial absorbance for all samples was zeroed with buffer only. Samples were heated at a rate of 1°C/min from 20 to 95°C. Samples were first denatured, then renatured and then denatured again. The T_m_ was calculated as the average of temperatures at which the heating and annealing curves intersected the average line calculated from the slopes of the upper and lower limits of the annealing curves.

### UVB irradiation

UVB irradiation was carried out at 4°C immediately after sample preparation or after storage at 4°C overnight or for days with similar results unless otherwise indicated. G-quadruplex samples (50 µM) were irradiated in a polyethylene microcentrifuge tube and irradiated on a bed of ice for 2.5 h at a distance of ∼1 cm from the UVB lamp unless otherwise indicated.

### NP1-coupled HPLC/mass spectrometry assay

Irradiated samples (100 μl of 50 μM DNA) were digested with 1 μl of 1 U/μl aqueous NP1 from *P. citrinum* (Sigma) and 1 μl of 10 mM ZnCl_2_ at 37°C for >36 h. The digestion products were separated by reverse-phase HPLC on an X-Bridge column (C18, 4.6 × 75 mm, 2.5 µm, 135-Å pore size, Waters Corporation) using a 1 ml/min of 100% solvent A (50 mM triethylammonium acetate, pH 7.5) for 3 min followed by a linear gradient of 0–20% B (50% acetonitrile in 50 mM triethylammonium acetate, pH 7.5) in solvent A for 3–53 min and detected at 260 nm. The yield of each photoproduct-containing digestion product was calculated from the integral of the corresponding HPLC peak at 260 nm by using molar extinction coefficients for the appended undamaged nucleotide or nucleotides at 260 nm relative to the integral for one of the undamaged nucleotides. The HPLC fractions corresponding to photoproduct-containing NP1 digestion fragments were dried *in vacuo*, and Milli-Q water was added to each sample to yield a final ODN concentration of ∼50 μM. One microliter of sample was mixed with 1 µl of 3-hydroxypicolinic acid with 10% ammonium citrate and spotted on a stainless-steel MALDI plate. MALDI MS spectra were taken in reflectron positive ion mode and were the average of 20 accumulations.

### Denaturing polyacrylamide gel electrophoresis experiments

Tel26 was incubated with 10 U of T4 polynucleotide kinase (Fermentas Life Science) and 20 pmol of [α-^32^P]-ATP at 37°C for 1 h in 500 mM Tris-HCl, pH 7.6, 100 mM MgCl_2_, 50 mM dithiothreitol, 1 mM spermidine and 1 mM ethylenediaminetetraacetic acid. The reaction was quenched by adding an equal volume of 2× loading buffer (98% formamide, 10 mM ethylenediaminetetraacetic acid), boiling for 2 min and cooled to room temperature. The radiolabeled sample was then analyzed following UVB irradiation on a 15% denaturing polyacrylamide gel and scanned with a Personal Molecular Imager (Bio-Rad Laboratories) and analyzed with Quantity One software. For large-scale isolation of photoproduct-containing Tel26 for CD spectroscopy, 50 μM was irradiated in 1 ml of 150 mM KCl for 2.5 h and then concentrated by evaporating solvent *in vacuo*. The sample was reconstituted in formamide loading buffer and electrophoresed in multiple lanes on a 15% denaturing polyacrylamide gel electrophoresis gel. The ODN-containing bands were visualized by brief exposure (<30 s) of the edges of the gel to 254-nm light against a fluorescent thin layer chromatography plate, and a razor was used to excise bands from the gel. The gel slices were eluted overnight at 65°C, and the DNA was recovered by standard ethanol precipitation and dissolved in 100 μl of 150 mM KCl Tris buffer for CD measurements.

## RESULTS

**Figure 2.**
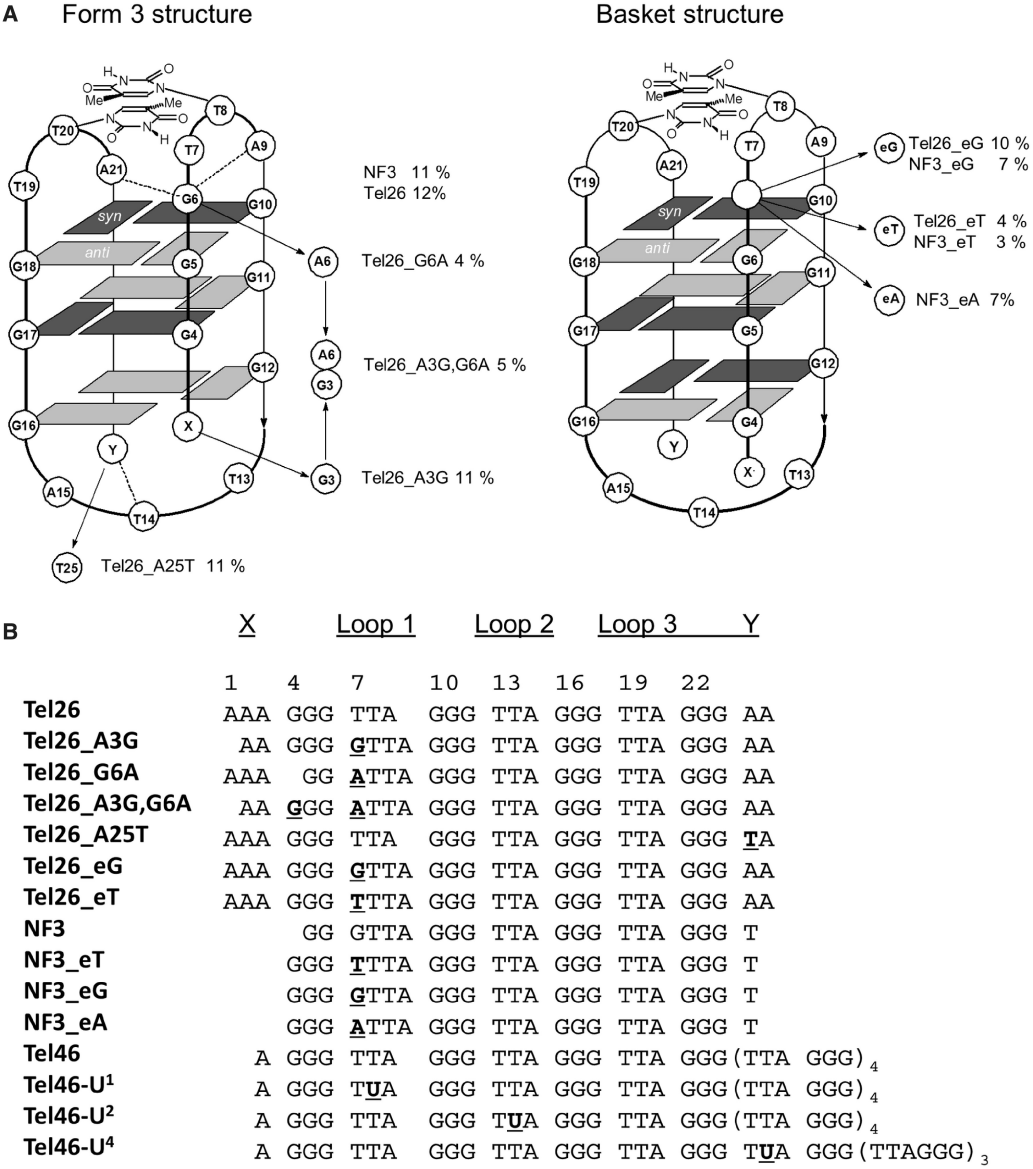
Human telomeric DNA sequences studied. (**A**) Locations of mutations in Tel26 and NF3 designed to test the involvement of the two-tetrad form 3 structure in *anti* CPD formation along with the yields of the *trans,anti* T(A)=T(A) photoproduct. Dashed lines indicate capping interactions. (**B**) Sequences studied with proposed loop assignments, and changes made to the parent structure indicated in bold underline.

To elucidate the structure–activity relationships of UV-induced *anti* CPD formation in human telomeric G-quadruplexes, we first focused on Tel26. The Tel26 sequence has been established by NMR to preferentially exist in the hybrid-1 structure in K^+^ solution, which structurally cannot lead to any *anti* thymine cyclobutane dimers on UV irradiation owing to the large spatial separation between the loops, which would prevent stacking of the two thymine bases. Despite this, Tel26 highly produces almost exclusively the *trans,anti* CPD of T(A)=T(A) on UVB irradiation, suggesting the involvement of a specific photoreactive intermediate structure. Examination of NMR structures of the basket structure for the Na^+^ form of Tel22 suggests that the Ts in loops 1 and 3, while in the correct *anti* orientation, may be too far apart to photodimerize. On the other hand, the Ts in the NMR structures of the form 3 structure in K^+^ solution appear to be able to overlap due to the presence of a 4-nt loop 1, although some rearrangement would be required to achieve the required *anti* orientation. The first goal of this study was to see whether the yield of the *trans,anti* CPD was enhanced in the NF3 sequence and whether *anti* CPD formation could be further enhanced by sequence modifications to Tel26 and NF3 (Figure 2) that would stabilize a 4-nt loop 1 basket structure with three G-tetrads in both Na^+^ and K^+^ solutions.

### Preparation and characterization of the telomeric G-quadruplexes

**Figure 3.**
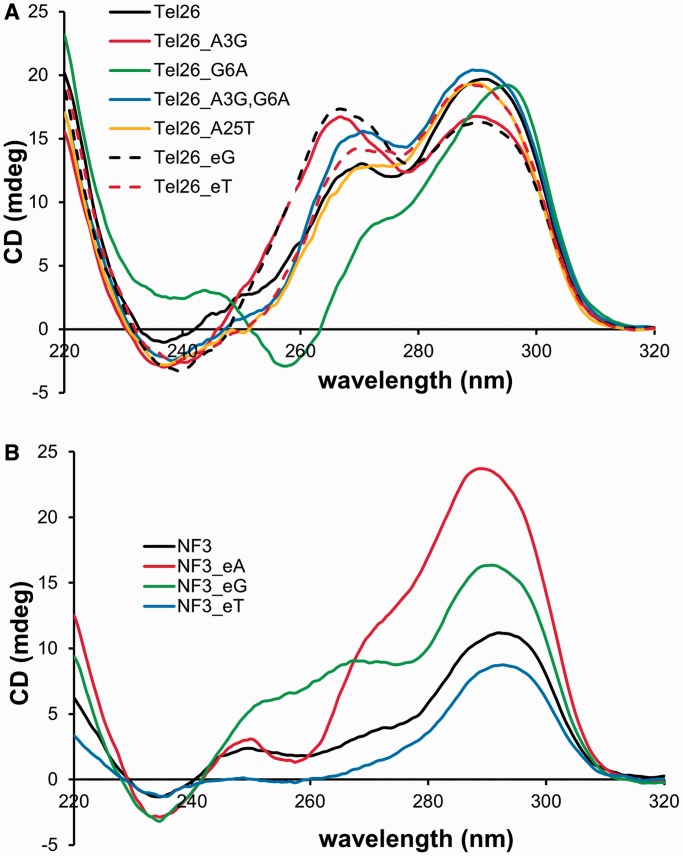
CD spectra of the (**A**) Tel26- and (**B**) NF3-derived sequences in K^+^ buffer at 4°C.

The G-quadruplexes were prepared from commercially synthesized ODNs by denaturing and then quickly cooling on ice. CD spectra of Tel26 and NF3, in both Na^+^ (Supplementary Figure S1) and K^+^ (Figure 3 and Supplementary Figure S2) solutions, closely matched the reported spectra (10–12). Positive CD peaks at 265 and 295 nm are generally taken as a sign of antiparallel G-quadruplex structure (11), though the peak at 265 becomes negative in the antiparallel basket structure in Na^+^ solution. The CD spectra of the Tel26 and NF3 sequences at room temperature were similar to those at 4°C, although the NF3 sequences showed differences in intensity (Supplementary Figure S2). In Na^+^ solution at 4°C, all of the Tel26 sequences had similar CD spectra from 280 to 320 nm, but showed significant differences between 240 and ∼260 nm (Supplementary Figure S1).

The melting temperatures of the quadruplexes in K^+^ solution (Supplementary Table S1) were determined by monitoring the absorbance at 295 nm from 20 to 95°C with a temperature ramp of 1°C/min in 150 mM KCl buffer. All sequences showed significant hysteresis (Supplementary Figure S3), suggesting that the rate of equilibration was slower than the heating rate (10,21). The mutations had minimal effect (±2°C) on the 64°C T_m_ of Tel26 except for Tel26_G6A, which was 28°C lower. All mutations of the NF3 sequence caused an average 5°C drop in T_m_ from 71°C. The melting temperatures in Na^+^ solution were not recorded.

### NP1-coupled HPLC assay of photoproduct formation in the G-quadruplexes

To determine the structure and yield of a particular photoproduct, an NP1-coupled HPLC assay was used (19,22). NP1 is an endonuclease that cleaves to the 3′ side of a nucleotide to produce a 3′-OH and a 5′-phosphate. Damaged bases that cannot be accommodated in the active site, such as the two thymines in a CPD, prevent phosphodiester cleavage immediately following either of the dimerized bases. In the case of an adjacent thymine dimer, such as a *cis-syn* thymine dimer, the trinucleotide pT=TN is produced (referred hereafter as T=TN), whereas with a nonadjacent thymine dimer, such as an *trans,anti* thymine dimer, the tetranucleotide, pT(N)=pT(N) [referred hereafter as T(N)=T(N)], is produced (Figure 1B). Tetranucleotides containing *anti* dimers can be readily distinguished from partial degradation products of undamaged DNA by having an 18-unit higher mass due the presence of a second terminal phosphate group instead of a phosphodiester [compare pN(N)=pN(N) with pNNNN].

**Figure 4.**
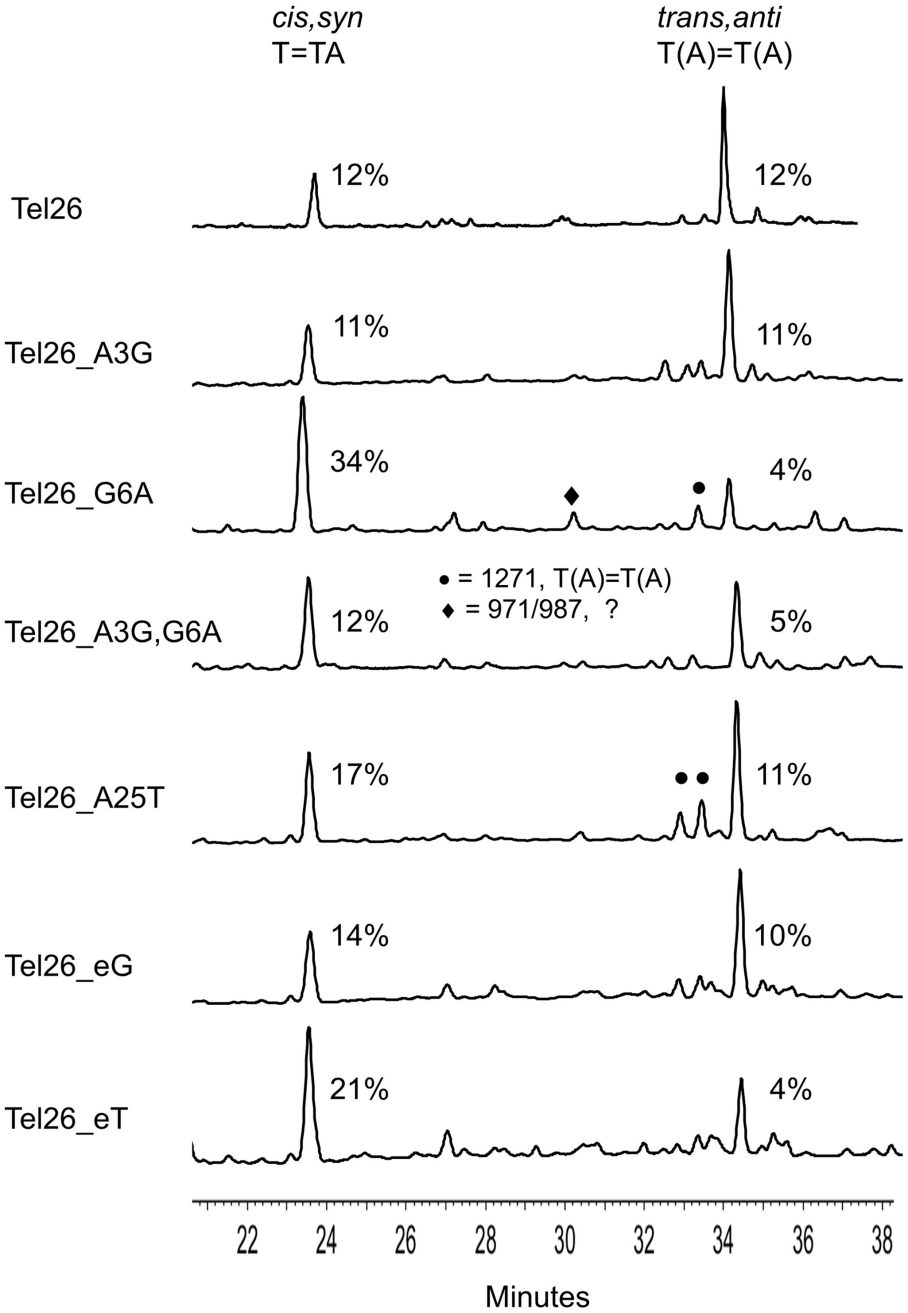
HPLC spectra of the nuclease P1 digestion products of the Tel26-derived sequences that had been irradiated for 2.5 h at 4°C in K^+^ buffer with UVB light under identical conditions.

**Figure 5.**
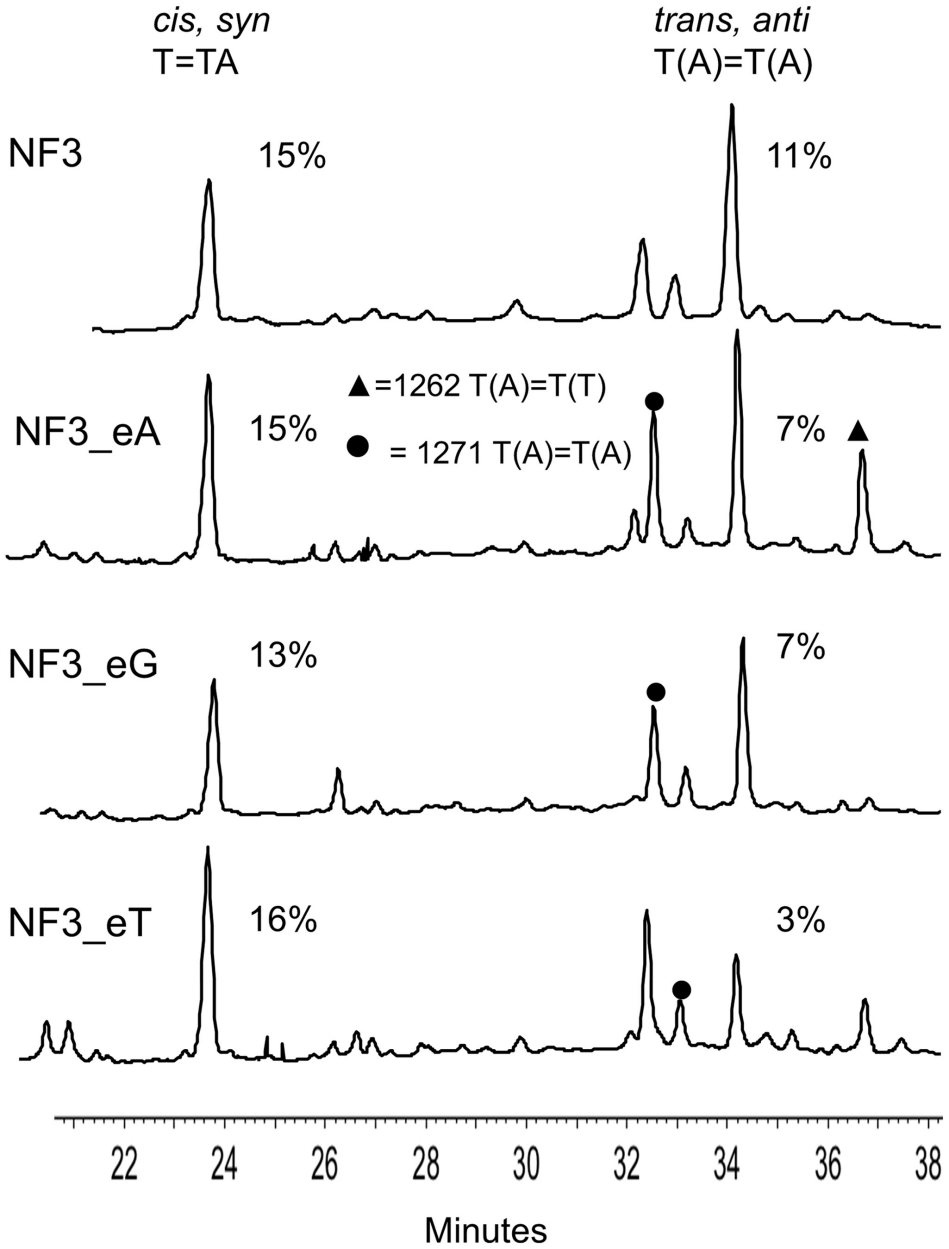
HPLC spectra of the nuclease P1 digestion products of the NF3-derived sequences that had been irradiated for 2.5 h at 4°C in K^+^ buffer with UVB light under identical conditions.

When the NP1 digestion products are analyzed by gradient reverse-phase HPLC (Figures 4 and 5), mononucleotides elute before 20 min, trimers elute between 20 and 30 min and tetramers elute between 30 and 40 min. MALDI was then used to confirm the nucleotide composition of the eluted photoproducts. The percentage yields of the photoproducts were calculated from the integrated absorbance of the HPLC peaks at 260 nm using estimated extinction coefficients for the photoproduct-containing tri- and tetranucleotides, relative to the actual ones for the mononucleotides. The advantage of the NP1-coupled HPLC assay compared with a glycosidic bond hydrolysis assay that liberates only the base portion (23,24) is that *anti* TT CPDs formed between three different pairs of Ts can be distinguished by mass according to the nucleotides appended to the *anti* thymidine CPD, i.e. T(T)=T(T), T(A)=T(A) and T(A)=T(T) (19). In addition, the 11 possible *cis,anti* and *trans,anti* CPD stereoisomers of these tetranucleotides can be separated by HPLC and their stereochemistries deduced by acid hydrolysis to yield the base portions (Figure 1C, R = H), which are compared to authentics (19,22). The analogous *anti* (6–4) photoproducts could also have been separated and detected by HPLC by virtue of their unique UV absorption at ∼320 nm, and by a unique fragment ion in their MS/MS spectra (25). These photoproducts were not detected in any significant amount, in this or the previous study (19), possibly because of their low quantum yield of formation and their facile photo isomerization to their Dewar valence isomers, which lack the 320-nm absorption peak (26) and which were also not detected.

### *Anti* CPD formation in Tel26 compared with NF3 in K^+^ solution

UVB irradiation of NF3, which had not been previously studied, in K^+^ solution gave essentially the same yield of *trans,anti* T(A)=T(A) CPD as did Tel26 in K^+^ solution (11%) (Figure 5). This was surprising, as NF3 primarily adopts the hypothesized photoreactive form 3 conformation in K^+^, whereas Tel26 adopts primarily the nonphotoreactive hybrid-1 conformation. Both sequences show distinctly different CD spectra in K^+^, as expected from their differing structures, with Tel26 showing two major peaks at ∼265 and 290 nm, and NF3 showing a greatly diminished 265 nm peak. Both sequences also had high melting temperatures of 64 and 71°C (Supplementary Figure S3). Irradiation of NF3 was less selective, however, producing two additional minor *anti* T(A)=T(A) CPDs that were identified by MALDI. These T(A)=T(A) CPDs appear to have the *cis,anti* stereochemistry based on comparison with products characterized in a previous study (19), and given the fact that there is only one possible *trans,anti* and two possible *cis,anti* T(A)=T(A) CPDs (27).

### Effect of increasing the loop size of the basket structure on *anti* CPD formation in K^+^ solution

To see whether *anti* CPD formation could be enhanced in K^+^ solution by increasing the size of loop 1 of Tel26 in a potential three-tetrad basket intermediate from three to four, an extra G, T or A was inserted into position 1 of loop 1. The introduction of C was not investigated because it would complicate the analysis by enabling the formation of CT CPDs. Adding the extra G (Tel26_eG) did not increase the yield of the *trans,anti* T(A)=T(A) CPD as expected, and instead lowered it from 12 to 10% (Figure 4). An extra G was also introduced into the first position of loop 1 by mutating A3 of Tel26 to a G (Tel26_A3G) to create a G tetramer that could slip up and force G6 into the loop. Irradiation of this sequence produced the *trans,anti* T(A)=T(A) CPD with the same yield as Tel26. In likewise manner, an extra A could be inserted into loop 1 by mutating A3 to G and G6 to A (Tel26_A3G,G6A), but this sequence gave the *anti* photoproduct in a lower yield of 5%. Adding an extra T (Tel26_eT) also decreased the yield to 4%, indicating the importance of having a G in the first position of loop 1, which forms a capping structure with A9 and A21 in the form 3 structure. With this mutation, an increase in the amount of *cis,syn* T=TA product was also observed. None of these Tel26 mutations affected the CD spectra or T_m_s (±2°C) in any significant way, suggesting that the structure and stability of the principal solution structures were not greatly affected.

### Effect of stabilizing the 4-nt loop 1 of the form 3 structure on *anti* CPD formation in K^+^

In this series of mutations, an extra G, A or T was inserted just before loop 1 of the NF3 sequence so as to increase the stability of the 4-nt loop conformation by enabling formation of a third G-tetrad. The sequence in which an extra G was inserted before loop 1 (NF3_eG) is similar to Tel26_eG, which has a G-tetramer that can slip down to form three G-tetrads while maintaining a 4-nt loop. The yield of *trans,anti* T(A)=T(A) CPD in this sequence, however, unexpectedly decreased to 7% (Figure 5) compared with 10% for Tel26_eG without greatly affecting the formation of two presumed *cis,anti* T(A)=T(A) CPDs. It may be that this sequence prefers to maintain a two-tetrad structure, and the extra G results in the formation of a 5-nt loop that inhibits CPD formation. Insertion of an extra A (NF3_eA) also gave the photoproduct in the same yield (7%), whereas an extra T (NF3_eT) reduced the yield to 3%, but without affecting the yield of the *anti* T(A)=T(A) CPDs. The decreased yield of the *trans,anti* CPD on insertion of A and T is consistent with destabilization of the A9•G•A21 capping structure in the form 3 structure (Figure 2A). However, the addition of A and T also caused the appearance of an *anti* T(A)=T(T) product identified by MALDI, which must presumably arise from a different conformation than that from which the *trans,anti* T(A)=T(A) product arises. None of the insertion mutations changed the overall shape of the CD spectra, except for NF3_eG, though there were differences in intensity between 4°C and room temperature, and the T_m_s only dropped by an average of 5°C. The 265 nm band in the CD spectrum of NF3_eG appeared to have increased in comparison with that of NF3 and was more similar to that of Tel26.

### Mutations probing the effect of form 3 capping structures on *anti* CPD formation in K^+^

To further probe the capping structures (Figure 2A), G6 in Tel26 was mutated to A (Tel26_G6A) so that Tel26 could only form a two-tetrad basket-like form 3 structure with a 4-nt loop in which the G at position 1 of the loop was changed to an A. Unlike all the other mutations of Tel26, this compound mutation caused a devastating drop in the T_m_ of 28°C compared with that of Tel26, and changed the CD spectrum from that of Tel26 to that of the form 3 structure. *Trans,anti* T(A)=T(A) formation in this sequence diminished to 4%, which was the same as observed for Tel26_A3G,G6A which would form the same 4-nt loop, further pointing to the possible importance of G in position 1 of loop 1 for forming the form 3 capping structure. Surprisingly, the yield of the *cis,syn* T=TA product jumped up from 12 to 34%, perhaps because destabilization of the G-quadruplex structure led to conformations much more favorable for adjacent CPD formation. It has also been reported that T25 of NF3 (numbering scheme in Figure 2A) is critical in stabilizing the form 3 conformation by hydrogen bonding to the nonadjacent T14 (13). Changing A25 in the Tel26 sequence to T (Tel26_A25T) would enable a similar interaction with T14, but irradiation of this sequence did not increase the yield of the *trans,anti* CPD compared with Tel26 but did lead to the formation of the same two minor *cis,anti* T(A)=T(A) photoproducts seen with the NF3 sequence.

### Isolation and characterization of photocrosslinked Tel26

**Figure 6.**
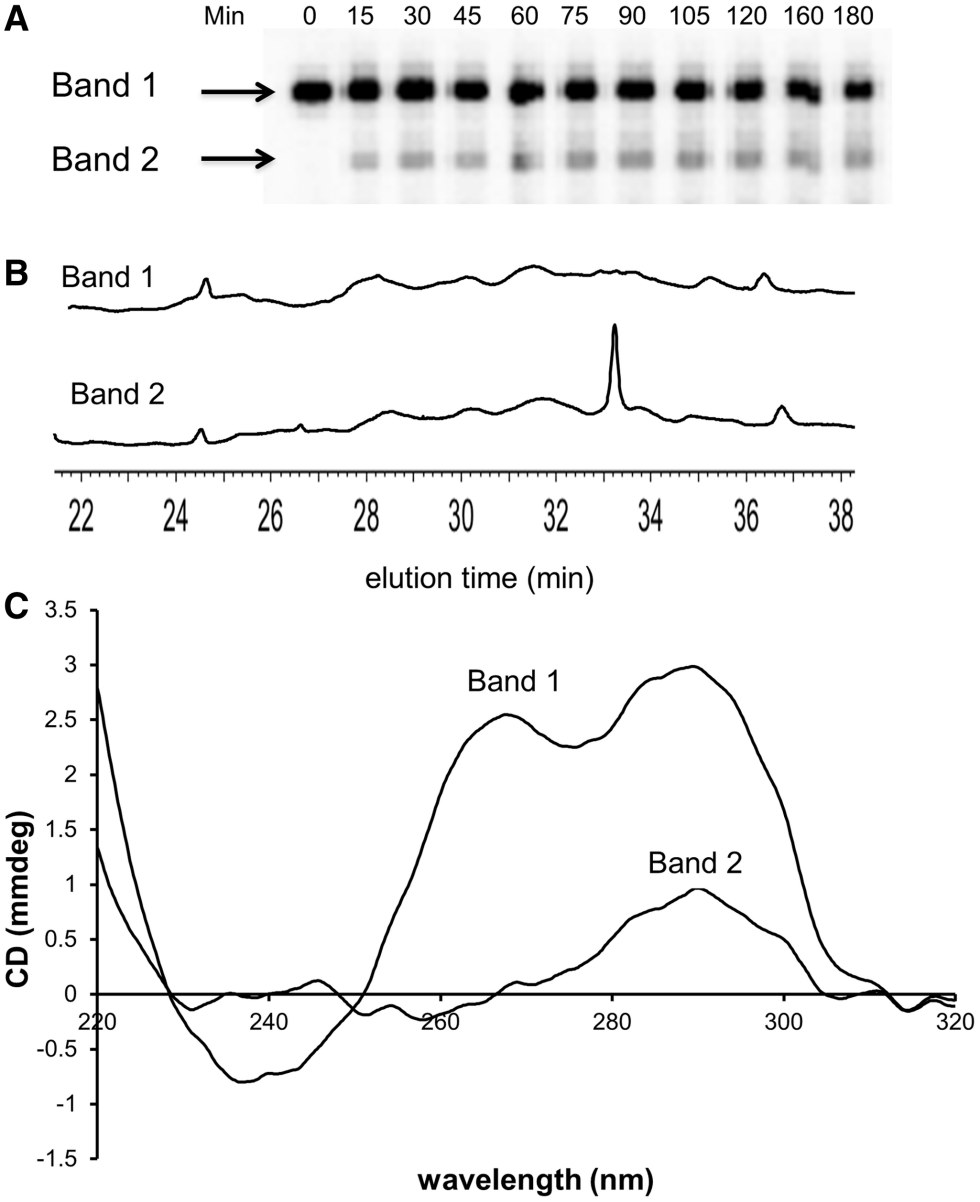
Isolation and CD spectrum of the *trans,anti* T(A)=T(A) containing Tel26. (**A**) Polyacrylamide gel electrophoresis of 5′-^32^P-end labeled Tel26 that had been irradiated with UVB for the indicated times at 4°C in K^+^ buffer. (**B**) Nuclease P1-coupled HPLC assay of Band 1 and Band 2 that had been irradiated for 2.5 h. (**C**) CD spectra of the ODNs isolated from Band 1 and Band 2.

Irradiation of Tel26 in K^+^ solution produced a faster moving band (Band 2) on denaturing polyacrylamide gel electrophoresis (Figure 6A) that was previously shown for Tel22 to contain the *anti* CPD photoproducts (19). NP1 digestion of the DNA eluted from Band 1 did not show significant presence of any photoproducts, whereas the DNA eluted from Band 2 showed the presence of the *trans,anti* T(A)=T(A) CPD (Figure 6B). The CD spectrum of the DNA from Band 1 was the same as that for Tel26, but the DNA from Band 2 had a CD spectrum with much greater similarity to that of NF3 (Figures 6C) suggesting that *trans,anti* CPD had locked Tel26 into the form 3 conformation.

### Kinetics of CPD formation in Tel26 and NF3

**Figure 7.**
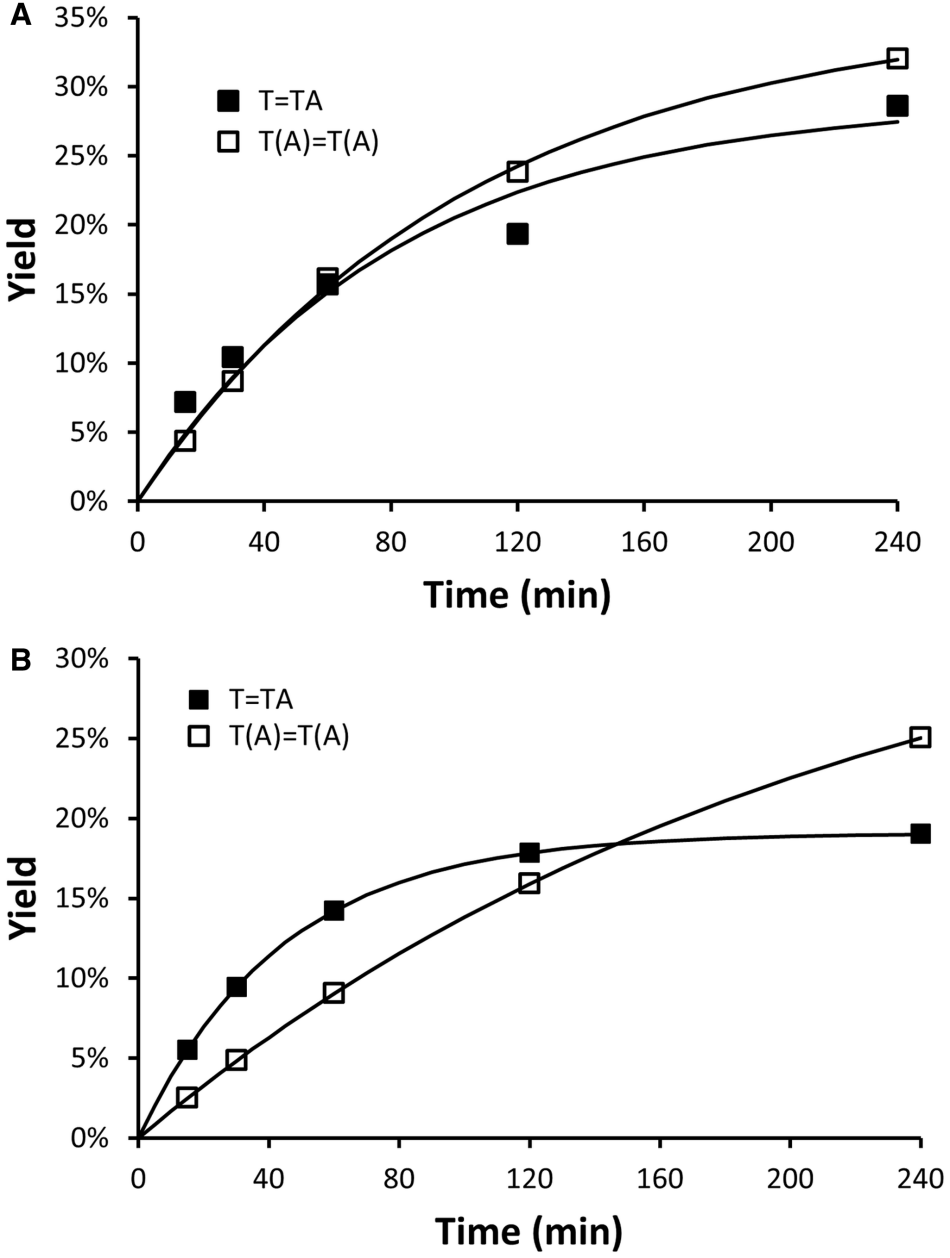
Kinetics of photoproduct formation in Tel26 and NF3. *Cis,syn* and *trans,anti* CPD formation in (**A**) Tel26 and (**B**) NF3 determined by the nuclease P1 assay. In this study, a higher intensity of UVB light than that used in the experiments in Figures 4–6 was used.

To gain more insight into the mechanism of *anti* CPD photoproduct formation, their rate of formation in Tel26 and NF3 was investigated by NP1 digestion as a function of UVB irradiation time (Figures 7A and B). For both sequences, *trans,anti* T(A)=T(A) photoproduct formation appeared to fit a simple first order process that was projected to approach a maximum of ∼35%. Surprisingly, the initial rate of formation of the *trans,anti* T(A)=T(A) CPD was about two times faster for Tel26 than for NF3 (0.0029 versus 0.0015 min^−^^1^), even though NF3 was proposed to adopt primarily the photoreactive form 3 conformation, whereas Tel26 was not. In comparison, the initial rate of *cis,syn* T=TA CPD formation was greater than *trans,anti* CPD formation for both Tel26 and NF3 (0.0047 min^−^^1^), but was projected to reach a lower maximum yield for NF3 than for Tel26 (19 versus 29%). It is worth noting that the maximum theoretical yield of T=TA per ODN is 3 compared with 1 for T(A)=T(A), as there are three independent T=TA photoproduct-forming sites per sequence, whereas only one T(A)=T(A) photoproduct can form per sequence.

One possible explanation for the limiting yield of *cis,syn* and *trans,anti* CPD formation is that it represents a steady state value resulting from competitive photoproduct reversal. To test this hypothesis, a sample of *trans,anti* T(A)=T(A) CPD-containing Tel26 was desalted and irradiated in 100 mM Na^+^ solution to prevent further photoproduct formation, but presumably allow for photoreversal. On irradiation for 30 min in Na^+^ solution, the *trans,anti* CPD appeared to increase in yield from 10 to 15%, but on further irradiation of an additional 30 and 60 min, no change in yield was observed, suggesting that photoreversal was not taking place, or at least not in Na^+^ solution (data not shown). It was also possible that oxidized Gs that might form during UVB irradiation could photocatalyze photoreversal of the cyclobutane thymine dimer, as has been previously observed 8-oxo-7,8-dihydroguanine (28). However, there was no evidence for the presence of 8-oxoGMP in the NP1 digestion samples by HPLC based on its distinct absorption maximum at 293 nm (29).

### Inhibitory effect of Na^+^ and higher temperature on *anti* CPD formation

The original hypothesis for why the *anti* photoproducts formed in low yield in Tel26 in Na^+^ solution was that Na^+^ was stabilizing the basket conformation, which only had 3-nt loops, and prevented formation of the form 3 structure with a two-tetrad basket and a 4-nt loop 1. Therefore, it was expected that adding an extra nucleotide to loop 1 of Tel26 or a third G to NF3 would result in a basket structure with a 4-nt loop 1, which would enhance *anti* CPD formation in Na^+^. Contrary to expectation, adding an extra nucleotide to loop 1 did not increase *anti* CPD formation compared with the parent Tel26 or NF3 in Na^+^ solution, although it did seem to alter the distribution of the products (Supplementary Figure S4).

**Figure 8.**
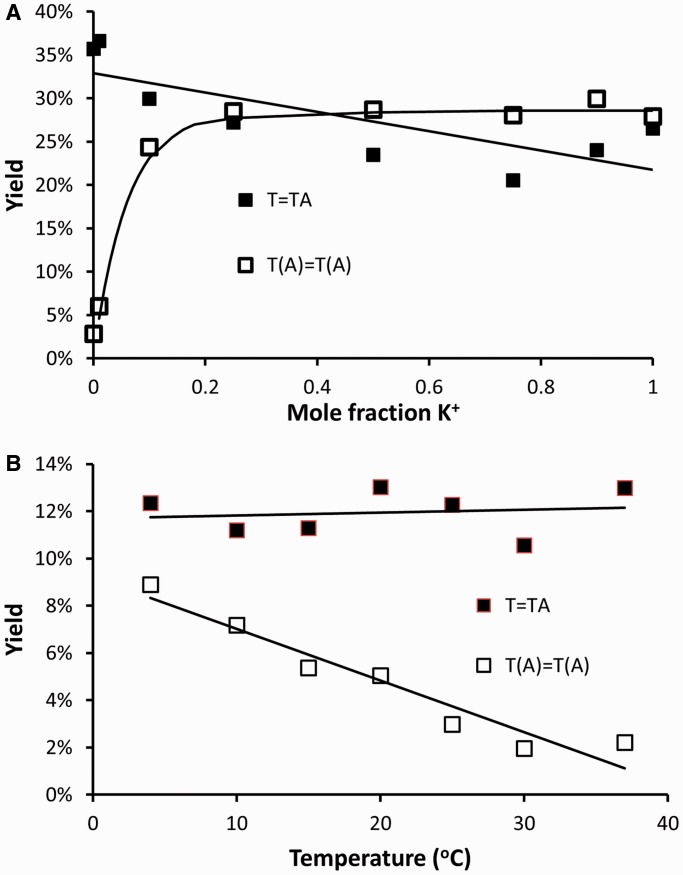
Effect of cation composition and temperature on *cis,syn* and *trans,anti* CPD formation. (**A**) Effect of the mole fraction of KCl in 100 mM NaCl on *cis,syn* T=TA and *trans,anti* T(A)=T(A) CPD formation using the same UVB intensity as in Figure 7 for 2 h. (**B**) Effect of temperature on *cis,syn* and *trans,anti* CPD formation in 150 mM KCl solution using the same intensity as in Figures 4–6 for 2 h.

To determine the relative ability of sodium to inhibit *anti* CPD formation, a competitive reaction was carried out between Na^+^ and K^+^ ions. To detect small changes in photoproduct yield, Tel26 was irradiated at a higher intensity in the presence of an increasing mole fraction of K^+^ in a 100 mM Na^+^ solution. As can be seen from Figure 8A, even a small concentration of K^+^ was sufficient to produce the same yield of *trans,anti* CPD as observed in 100 mM potassium solution, with a half maximal yield being observed at a concentration of 3.6 mM K^+^. The yield of *cis,syn* CPD decreased slightly on going from 100 mM Na^+^ to 100 mM K^+^ probably in part due to competition with *trans,anti* CPD formation, which would remove two sites for *cis,syn* CPD formation.

To determine the effect of temperature on the yield of the *trans,anti* CPD formation under more biologically relevant buffer conditions, Tel26 was irradiated from 4 to 37°C in 100 mM KCl, 1 mM MgCl_2_ in pH 7 buffer (Figure 8B). Although temperature did not have a significant effect on *cis,syn* CPD formation, which remained relatively constant at ∼11%, it had a much greater effect on *trans,anti* CPD formation, reducing the yield from ∼10% at 4°C to ∼1% at 37°C.

### Photoproduct formation in tandem human telomeric G-quadruplexes

**Figure 9.**
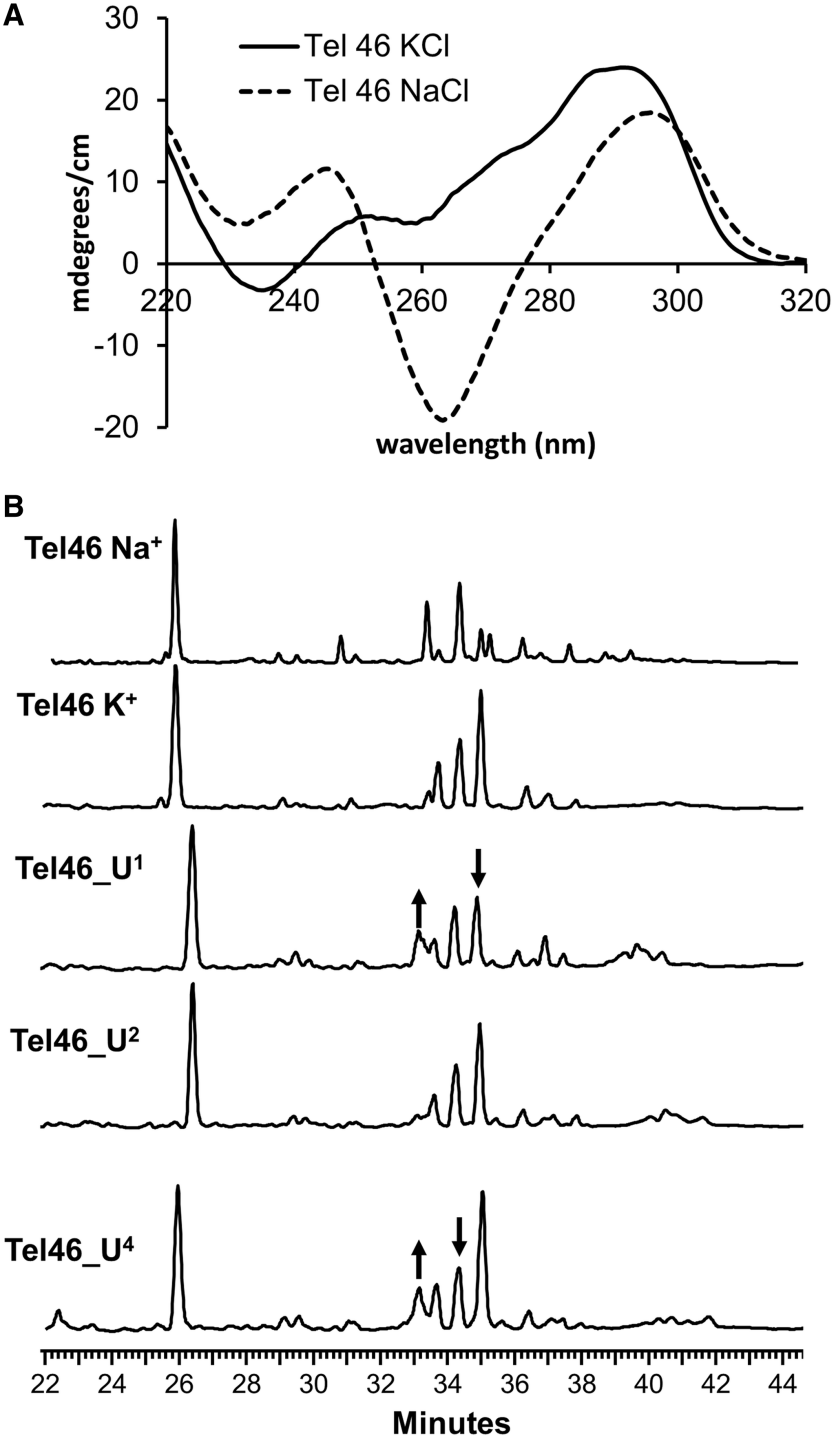
Photoproduct formation in a tandem G-quadruplex sequence. (**A**) CD spectra of Tel46 in 150 mM NaCl and KCl solution. (**B**) NP1 digestion products of UVB-irradiated Tel46 in 150 mM NaCl solution (top trace) and 150 mM KCl solution (bottom four traces) using the same intensity of UVB light as in Figures 4–6 for 2.5 h. Replacement of specific thymines with uracil in the Tel46 sequence shows that while the thymine in loop 2 (Tel46-U^2^) does not participate in the formation of *anti* thymine dimer, the thymine in both loops 1 and 4 do (Tel46-U^1^ and Tel46-U^4^). Arrows indicate changes in the relative amounts of photoproducts compared with the native sequence. The experiments were run twice, and owing to variations in the elution times, the spectra were aligned with the tetramer products for comparison purposes.

To determine whether *anti* CPDs might also form in longer telomeric tracts, a 46-nt long human telomeric sequence containing two G-quadruplex forming sequences, Tel46, was irradiated with UVB light in K^+^ and Na^+^ solutions. The CD spectra of Tel46 in Na^+^ and K^+^ solutions are shown in Figure 9A. In K^+^-containing solution, the predominant species appears to be the two-tetrad form 3 basket conformer as indicated by the small peak at 252 nm and the large peak at 293 nm. In contrast, Tel46 in Na^+^ solution shows the characteristics of the basket conformation with a large negative peak at 263 nm. In K^+^ solution, the *trans,anti* T(A)=T(A) was the major product along with two other *anti* products, while in Na^+^ solution, the amount of *trans,anti* T(A)=T(A) CPD was greatly reduced, though other *anti* products were observed (Figure 9B). To identify some of the *anti* CPD sites, uracil was site-specifically substituted for the second T in loops 1, 2 and 4 of Tel46. Uracil can undergo the same photo [2 + 2] cycloaddition reactions as thymine to give a *syn* or *anti* CPD, but results in a product with a different retention time after NP1 digestion because of the replacement of the hydrophobic methyl group with a hydrogen. Similar NP1 digest products were seen for both Tel46 and Tel46-U^2^, suggesting that the second thymine in loop 2 does not participate in loop photocrosslinking. However, the pattern of NP1 digestion products changed when uracil was substituted in either loops 1 (Tel46-U^1^) or 4 (Tel46-U^4^). For loop 1, the *trans,anti* T(A)=T(A) CPD peak appeared to diminish, and a new peak at 33 min appeared, whereas for loop 4, the peak at 34.5 min appeared to diminish while the same new peak at 33 min appeared.

## DISCUSSION

Evidence for *anti* CPD formation in DNA was first obtained for duplex DNA that was either dehydrated or was in the presence of alcohol (30). A *cis,anti* CPD was later accidentally discovered to form between Ts at positions 2 and 7 during UVB irradiation of a single-stranded 14-mer at pH 5 in aqueous solution, for which no obvious secondary structure would have been predicted (22). This latter discovery led to a search for *anti* CPD formation in human telomeric DNA, which was expected to facilitate *anti* CPD formation between adjacent lateral loops in certain G-quadruplex structures. Surprisingly, the *anti* dimer was not found to form efficiently in the basket-type structure established by NMR for Tel22 in Na^+^ solution (9), but did form in K^+^ solution, which favors hybrid structures (10,11) that would not allow for *anti* CPD formation. At that time, an alternate two-tetrad basket structure (form 3) was discovered for the human telomeric NF3 sequence, which was related to the basket structure in Na^+^ solution except that one of the strands slips to result in a larger 4-nt loop at the expense of one of the G-quartets (12). Therefore, it was proposed that the larger loop 1 in the two-tetrad form 3 structure would enable *anti* CPD formation in K^+^ solution. To account for why *anti* CPDs were suppressed in Na^+^ solution, it was proposed that Na^+^ was stabilizing the three-tetrad basket structure with 3-nt loops that would prevent *anti* CPD formation. To test thisproposal, the photochemistry of the NF3 sequence and mutants of the Tel26 and NF3 sequences have been studied.

The first surprising result was that the NF3 sequence, which primarily adopts the proposed photoreactive form 3 structure, while leading to the expected *trans,anti* T(A)=T(A) CPD, did so in no greater yield than did Tel26, which primarily adopts the nonphotoreactive hybrid-1 structure in K^+^ solution (10) (Figures 4 and 5). This suggests that the two-tetrad form 3 conformation is not the photoreactive one, or the yield should have been much greater. In addition, the photochemistry was less selective for NF3 than for Tel26, resulting in what appears to be the two possible *cis,anti* isomers of the T(A)=T(A) CPD, suggesting that the two sequences must be accessing different photoreactive conformations. While the photoreactive conformation may not be the two-tetrad form 3 conformation, the similarity of the CD spectrum of Tel26 containing the *trans,anti* T(A)=T(A) CPD to that of NF3 (Figure 6C) suggests, however, that the photocrosslinked product may adopt that conformation.

Given that there was no obvious correlation between what has been established as being the major structure for Tel26 and NF3 in K^+^ solution and the yield of the *trans,anti* CPD*,* it is possible that the reactive conformations are only present as minor components that are in equilibrium with other conformations. In addition, it appears that certain changes in the sequence of Tel26 or NF3, such as the addition of A or T into loop 1 can cause changes in the yield and ratio of photoproducts produced, presumably by affecting the relative stability of the photoreactive conformations, while other changes do not. Human telomeric ODNs have been shown to be in equilibrium with a variety of conformations by force jump experiments (31). When the kinetics of *trans,anti* T(A)=T(A) CPD formation was investigated, it was found that Tel26 produces the *trans,anti* T(A)=T(A) CPD at twice the initial rate of NF3 (Figure 7). This result again suggests that the two-tetrad form 3 conformation is not the photoreactive one but that a related conformation or intermediate is involved, otherwise the rate should have been faster for the NF3 sequence. It is not clear at this moment as to what this conformation or intermediate is. In our previous study, both a truncated and mutated Tel22 sequence that were only capable of forming a triplex structure showed greatly diminished yields of *anti* CPDs, suggesting that triplex structures were not involved (19). One possibility is that the chair form is involved (Supplementary Figure S5). The chair form is the only other type of G-quadruplex structure that has adjacent lateral loops capable of forming an *anti* CPD. It only differs from the basket structure in having a lateral loop in place of the diagonal loop, and has been detected in human telomeric sequences (32). Another possibility is that the hairpin intermediate leading to the chair form is involved (Supplementary Figure S5) (33).

Whatever the photoreactive conformations are in K^+^ solution, they also appear to be affected by temperature. When Tel26 is warmed to 37°C in a buffer with an intracellular-like salt composition of 150 mM K^+^ and 1 mM Mg^2+^, the yield of the *trans,anti* T(A)=T(A) photoproduct decreased, while that of the *cis,syn* T=TA CPD did not (Figure 8B). This did not seem to be directly related to the average conformation, as the CD spectra at 4°C and room temperature were similar (Figure 3 and Supplementary Figure S2), though one cannot rule out that the fraction of a small amount of photoreactive conformation was decreasing with increasing temperature. The decrease in *trans,anti* T(A)=T(A) might also be explained by a decrease in the lifetime of the photoreactive conformations leading to the *trans,anti* CPD relative to the excited state lifetime as the temperature increases. The formation of the *cis,syn* CPD would not be expected to be as affected by temperature because the two Ts undergoing the photoreaction are always adjacent to each other.

The second surprising observation was that Na^+^ still suppressed *anti* CPD formation in sequences that should have been able to form the same 4-nt loop 1 basket structure in Na^+^ solution that was hypothesized to enable the *anti* CPD to form in K^+^ solution (Supplementary Figure S4). One possible explanation is that Na^+^ holds one of the Ts in the loops of the G-quadruplex tightly in a nonphotoreactive conformation and prevents it from adopting a photoreactive conformation. In support of this idea, it had been proposed that differences in the loop structures in Oxytricha G-quartet (G_4_T_4_G_4_) observed in Na^+^ and K^+^ solutions were due to the manner in which these metal ions bind to the G tetrads (34). It was suggested that because Na^+^ binds in the plane of the terminal G-tetrad, it is able to coordinate to the carbonyl oxygen of thymine, whereas K^+^ cannot because it is held between two G-tetrad planes. Photoreactivity was restored on the addition of even a small proportion (3%) of K^+^ ion to the Na^+^ solution (Figure 8A), which is in accord with the higher binding affinity of K^+^ than Na^+^ for G-quadruplex structures (35–37). An alternate explanation that the *anti* CPDs were being photoreversed in Na^+^ solution was ruled out by the failure to observe any photoreversal of *trans,anti* CPD-containing Tel26 in Na^+^ solution.

If Na^+^ coordination to a T explains the lack of photoreactivity of the basket structure with 3- or 4-nt loops, then there would be no need to invoke a 4-nt loop 1 containing basket structure as the principal photoreactive conformation in K^+^ solution leading to the *trans,anti* CPD. If so, all the data would be most consistent with the basket (Figure 2), chair or hairpin (Supplementary Figure S5) structures with 3-nt loops as the principal photoreactive conformations leading to the *trans,anti* T(A)=T(A) CPD. In accord with this, all the mutations that were designed to enforce a 4-nt loop 1 by the insertion of either A or T caused a drop in the yield of *trans,anti* CPD to ∼3–7%. In contrast, all the mutations that inserted a G, however, could still form a 3-nt loop and showed high yields of photoproduct (7–12%). The precise yield and photoproduct distribution would also depend on the actual sequence and its effects on the stability of other photoreactive and nonphotoreactive conformations as seen for the various Tel26 and NF3 mutants.

*Anti* CPD photoproducts were also observed to be formed in a longer telomeric sequence, Tel46, which contains two tandem G-quadruplex forming sequences. It is not surprising that the thymine in loop 1 of Tel46 appears to participate in *anti* CPD formation, as this is the position of *anti* CPD formation in Tel26. However, it is interesting that the thymine in loop 4 also appears to be involved in *anti* CPD formation, as according to the bead-on-a-string model, loop 4 is likely to serve as the linker between the two G-quadruplexes. If Tel46 were able to adopt multiple single G-quadruplex conformations, then one would have expected some fraction of loop 2 to have been involved in *anti* CPD formation, but this was not observed. Therefore, it is most likely that Tel46 is equilibrating between tandem G-quadruplex structures that can facilitate *anti* dimer formation between a loop and a linker. More studies will be needed with site-specifically labeled thymines to determine precisely where the *anti* thymine dimers are forming.

The results of this study demonstrate that *anti* CPD formation can occur under physiologically relevant conditions with biologically relevant UVB light, though the stereochemistry and yield of the *anti* CPDs produced depends highly on the sequence of the DNA, the cations present and the temperature. While the two-tetrad basket type conformation was originally proposed to be the photoreactive conformation in K^+^ solution, it appears that another, less stable conformation, such as the three-tetrad basket, chair or hairpin, may be involved. Although the yield of the *trans,anti* CPD appears to be diminished at higher temperatures, presumably because of rapid equilibration between temperature-dependent conformations, the quadruplex structures present in telomeres or promoters may be more constrained and less dynamic *in vivo*. Also, the skin, which is the principle target for UV-induced photoproducts, can be at much lower temperature than 37°C, especially in the winter, where people’s faces might be exposed to significant amounts of UV light when engaging in such activities as alpine skiing and mountaineering (38). Because G-quadruplex DNA has been shown to inhibit cellular replication and transcription (39–41), photocrosslinking of these structures through *anti* CPD formation would be expected to enhance these inhibitory effects. Clearly, further research on the formation, repair and replication of *anti* CPD formation in other G-quadruplex structures *in vitro* and *in vivo* is needed to better understand the structure-activity properties of this novel class of photoproducts.

## SUPPLEMENTARY DATA

Supplementary Data are available at NAR Online.

## FUNDING

Research reported in this publication was supported by the National Cancer Institute of the National Institutes of Health under award number R01CA40463. Funding for open access charge: National Institutes of Health [NIH R01CA40463 to J.S.T.].

*Conflict of interest statement.* The content is solely the responsibility of the authors and does not necessarily represent the official views of the National Institutes of Health.

## Supplementary Material

Supplementary Data
