# Supplementary material for: Effect of sequence and metal ions on UVB-induced anti cyclobutane pyrimidine dimer formation in human telomeric DNA sequences
Source: Nucleic Acids Res. 2014 Mar 4;42(8):5007–19. doi: 10.1093/nar/gku163 (PMC4005637; doi:10.1093/nar/gku163)
Supplement: Supplementary Data [file supp_42_8_5007__index.html]

Effect of sequence and metal ions on UVB-induced anti cyclobutane pyrimidine dimer formation in human telomeric DNA sequences — Effect of sequence and metal ions on UVB-induced anti cyclobutane pyrimidine dimer formation in human telomeric DNA sequences — Supplementary Data 

# Effect of sequence and metal ions on UVB-induced *anti* cyclobutane pyrimidine dimer formation in human telomeric DNA sequences

## Supplementary Data

files

**Files in this Data Supplement:**

- Supplementary Data - pdf file
