# Supplementary material for: Effect of sequence and metal ions on UVB-induced anti cyclobutane pyrimidine dimer formation in human telomeric DNA sequences
Source: Nucleic Acids Res. 2014 Mar 4;42(8):5007–19. doi: 10.1093/nar/gku163 (PMC4005637; doi:10.1093/nar/gku163)
Supplement: Supplementary Data [file supp_gku163_nar-03260-f-2013-File011.pdf]

Supplementary Information

Effect of sequence and metal ions on UVB-induced *anti* cyclobutane pyrimidine dimer formation in human telomeric DNA sequences.

Jillian E. Smith,[1] Chen Lu, and John-Stephen Taylor*

Department of Chemistry, Washington University, St. Louis, MO 63130

* To whom correspondence should be addressed. Tel: +1 314 935 6721; Fax: +1 314 935 4481; Email: taylor@wustl.edu

[1]Present Address: Department of Chemistry, Emory University, Atlanta, GA, 30322, USA

Contents	Page
Table S1. Melting temperatures of the G-quadruplexes in 150 mM KCl.	S2
Figure S1. CD spectra of Tel26 and NF3 in 150 NaCl at 4°C and RT.	S3
Figure S2. CD spectra of Tel26 and NF3 in 150 mM KCl at RT.	S4
Figure S3. UV melting temperature curves for Tel26 and NF3 in 150 mM KCl.	S5-6
Figure S4. Photoproduct formation in Tel26 in 150 mM NaCl at RT.	S7
Figure S5. Possible hairpin and chair conformations of the Tel26 and NF3 sequences.	S8

Table S1. Melting temperatures of the G-quadruplexes in 150 mM KCl.

Sequence	average T_m (°C)	heating T_m (°C)	cooling T_m (°C)
Tel26	63.7	65.0	62.4
Tel26_A3G	65.7	66.8	64.6
Tel26_G6A	36.3	37.5	35.0
Tel26_A3G,G6A	62.5	63.9	61.0
Tel26_T25	63.5	64.5	62.5
Tel26_eG	64.5	66.0	63.0
Tel26_eT	63.0	64.0	62.0
NF3	71.1	72.0	70.1
NF3_eA	65.3	66.2	64.3
NF3_eG	66.4	67.4	65.4
NF3_eT	67.5	68.8	66.2

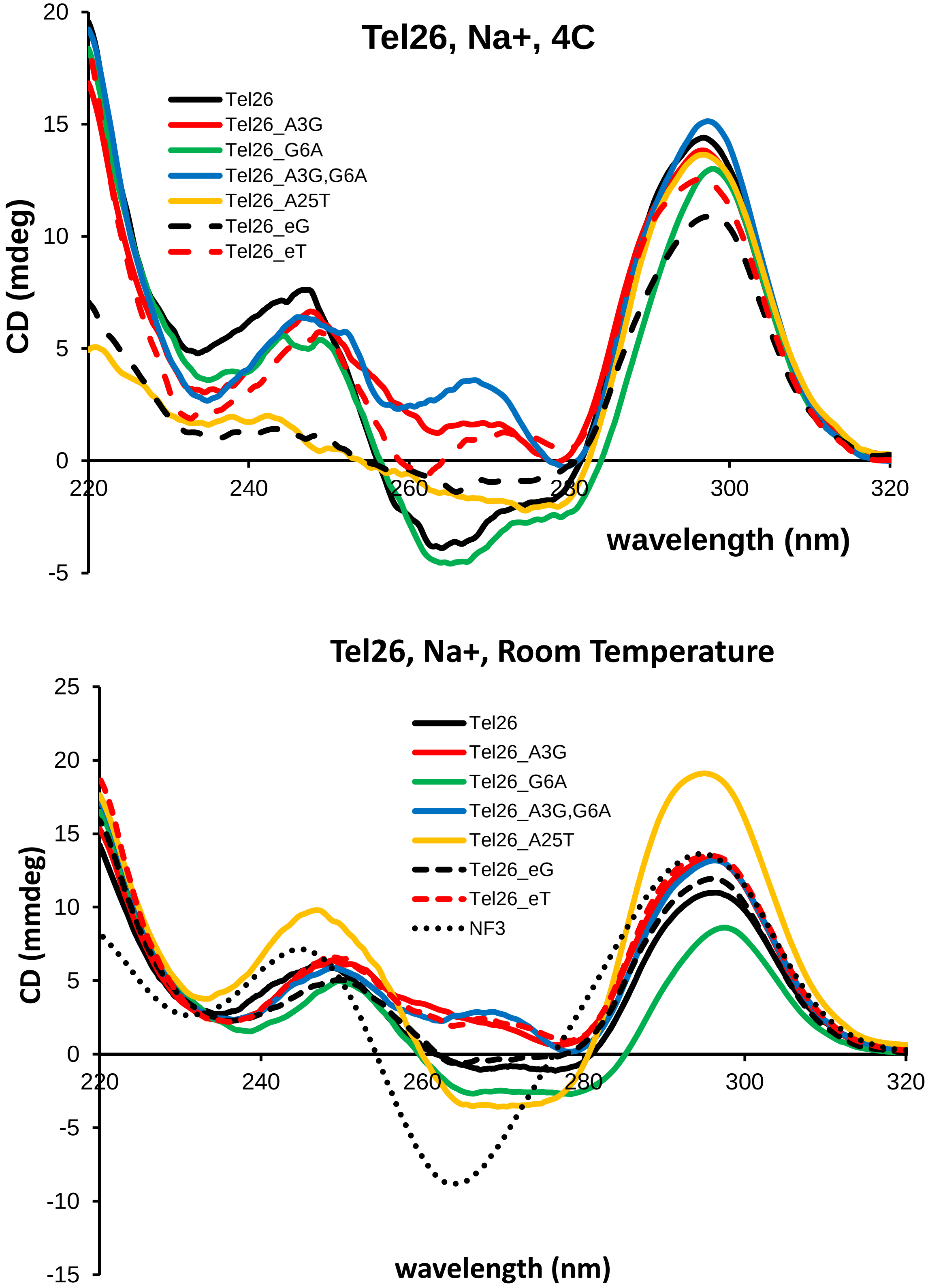

Figure S1. CD spectra of Tel26 and NF3 in 150 NaCl at 4°C and RT.

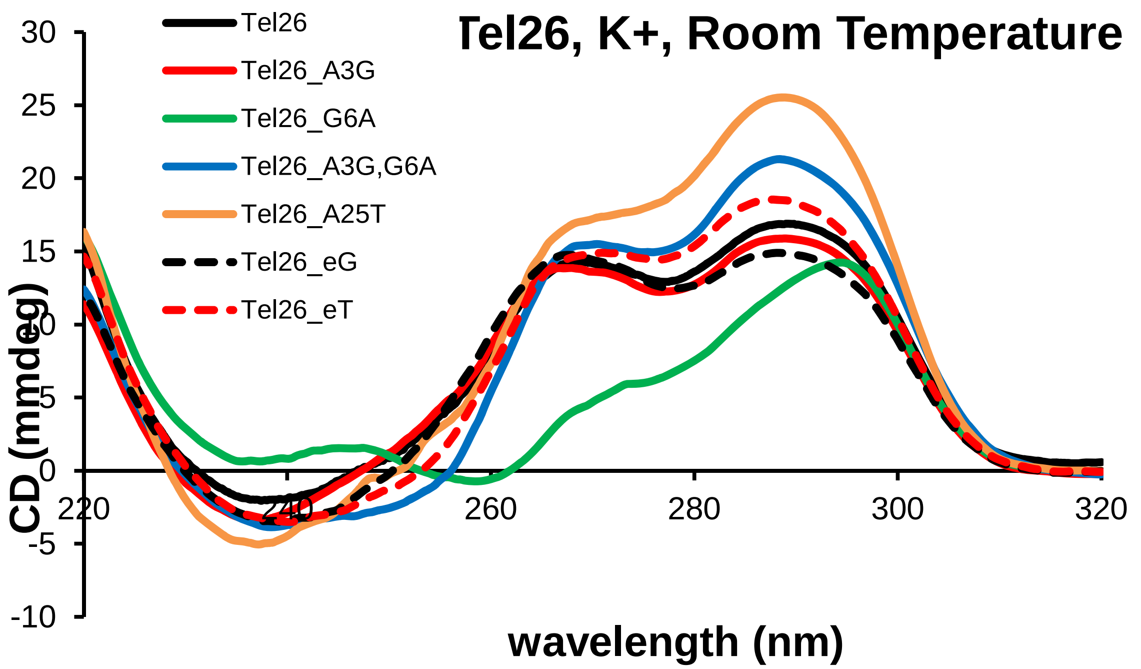

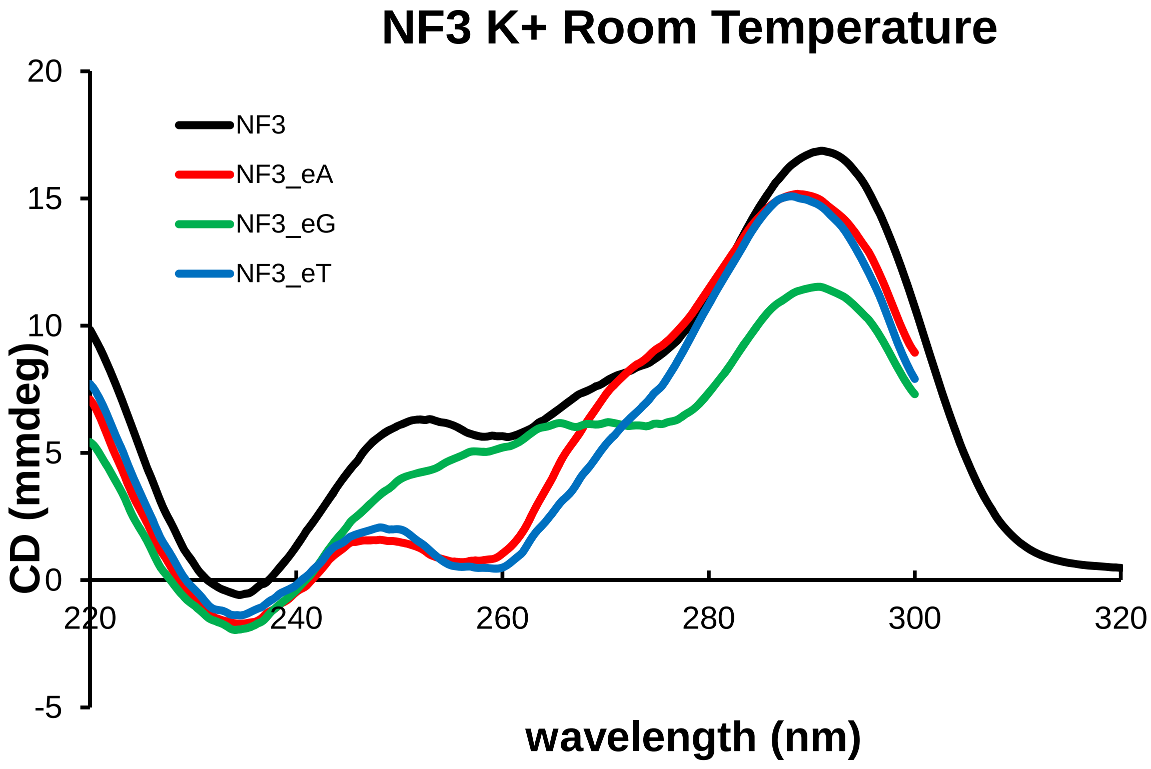

Figure S2. CD spectra of Tel26 and NF3 in 150 mM KCl at RT.

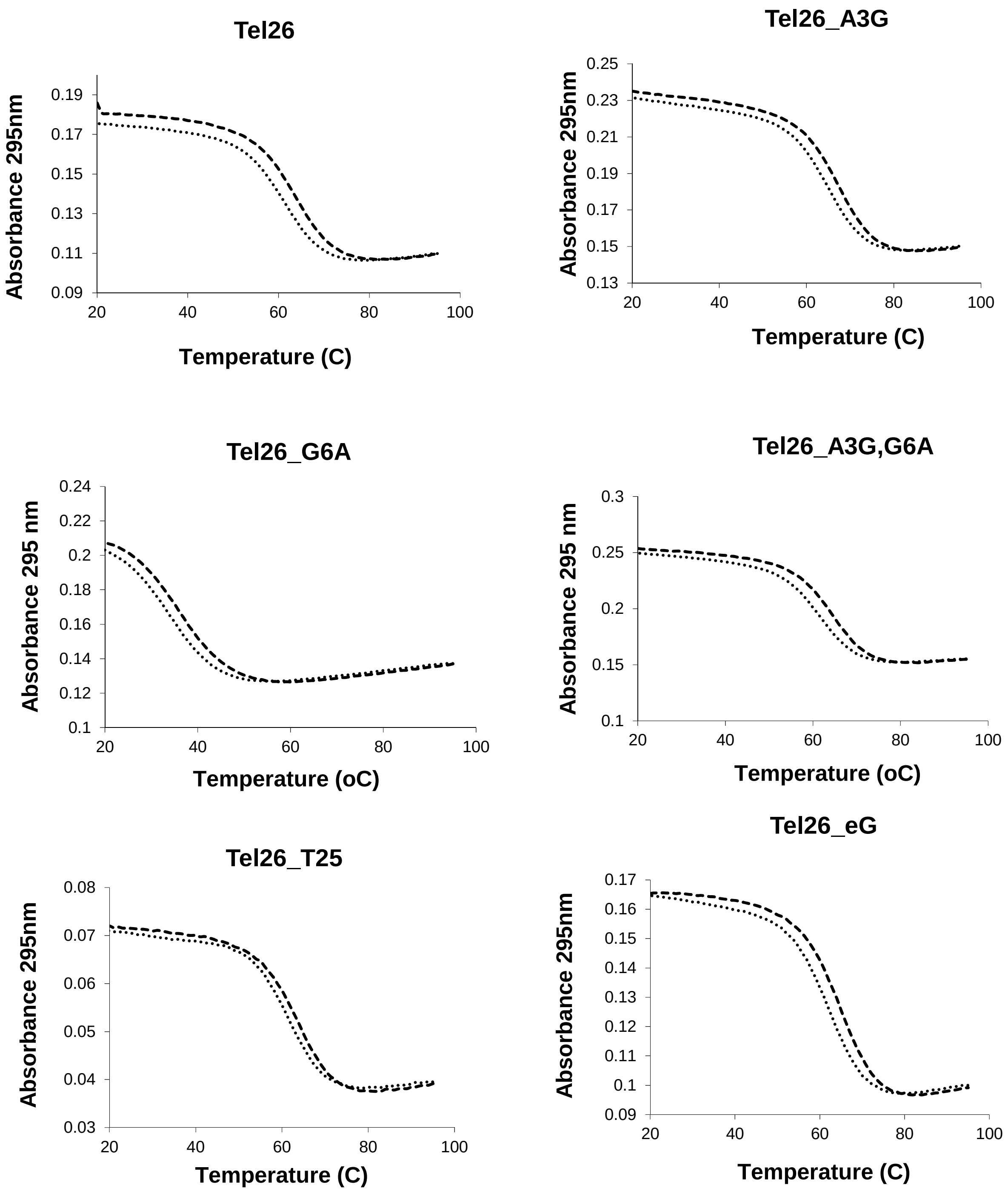

Figure S3. UV melting temperature curves for Tel26 and NF3 in 150 mM K^+ solution (continued next page).

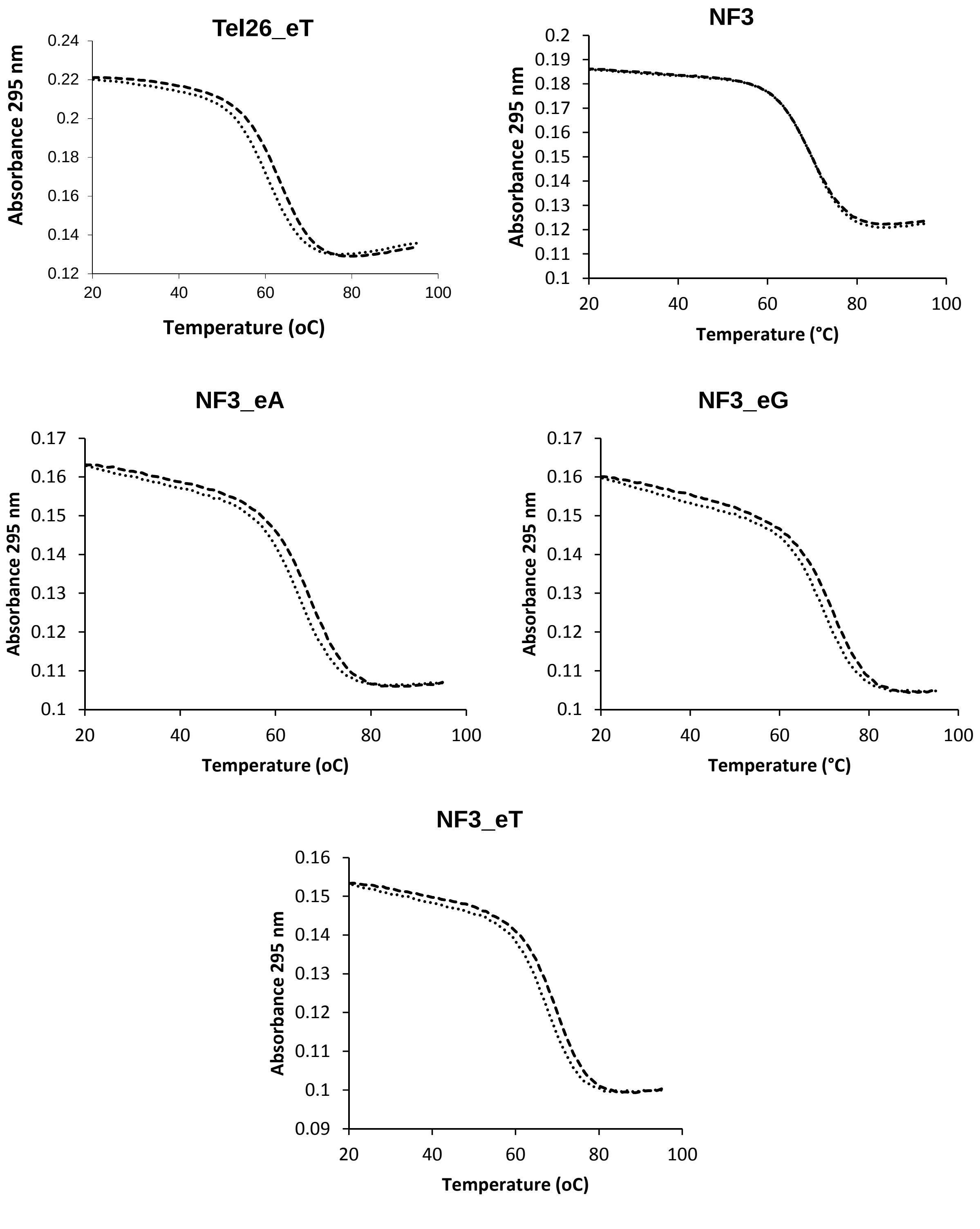

Figure S3 (continued). UV melting temperature curves for Tel26 and NF3 in 150 mM K^+ solution.

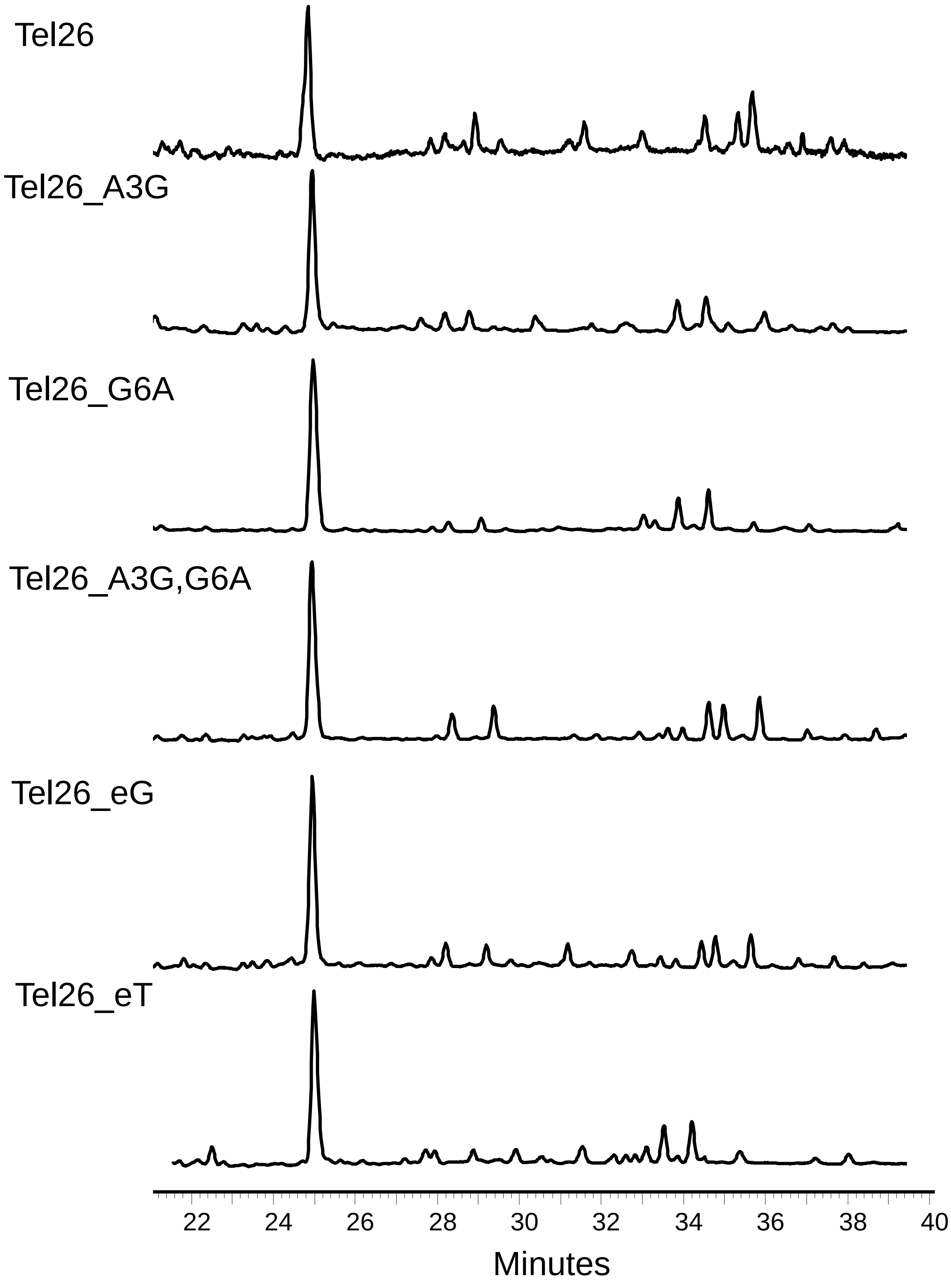

Figure S4. Photoproduct formation in Tel26 in 150 mM NaCl at 4°C.

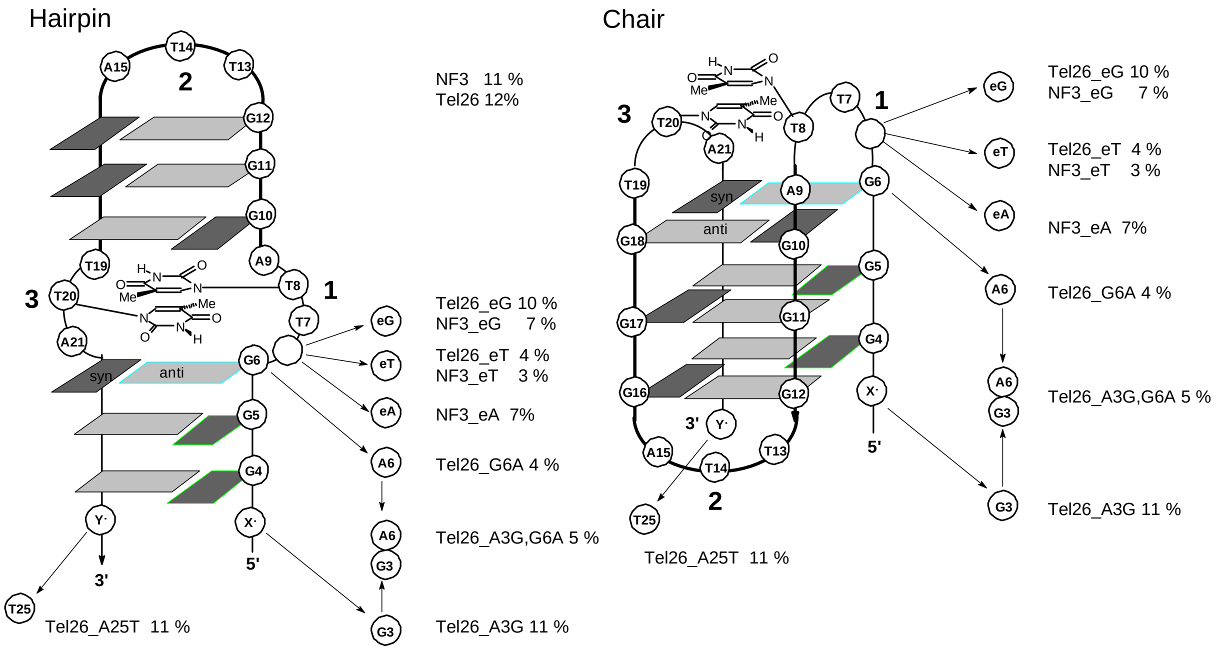

Figure S5. Possible hairpin and chair conformations of the Tel26 and NF3 sequences. The yields for each mutant are also consistent with photoreactive conformations that have two 3-nucleotide lateral loops.
